# Prefrontal Cortex and Supplementary Motor Area Activation During Robot-Assisted Weight-Supported Over-Ground Walking in Young Neurological Patients: A Pilot fNIRS Study

**DOI:** 10.3389/fresc.2021.788087

**Published:** 2021-12-10

**Authors:** Hubertus J. A. van Hedel, Agata Bulloni, Anja Gut

**Affiliations:** ^1^Swiss Children's Rehab, University Children's Hospital Zurich, Affoltern am Albis, Switzerland; ^2^Children's Research Center, University Children's Hospital Zurich, University of Zurich, Zurich, Switzerland; ^3^Department of Health Sciences and Technology, Institute for Human Movement Sciences and Sport, ETH Zurich, Zurich, Switzerland

**Keywords:** neuroplasticity, functional near infrared spectroscopy, pediatric neurorehabilitation, Andago®, supplementary motor area, prefrontal cortex, practicability, acceptability

## Abstract

**Introduction:** Rehabilitation therapy devices are designed for practicing intensively task-specific exercises inducing long-term neuroplastic changes underlying improved functional outcome. The Andago enables over-ground walking with bodyweight support requiring relatively high cognitive demands. In this study, we investigated whether we could identify children and adolescents with neurological gait impairments who show increased hemodynamic responses of the supplementary motor area (SMA) or prefrontal cortex (PFC) measured with functional near-infrared spectroscopy (fNIRS) when walking in Andago compared to walking on a treadmill. We further assessed the practicability and acceptability of fNIRS.

**Methods:** Thirteen participants (two girls, 11 boys, age 8.0–15.7 years) with neurological impairments walked in the Andago and on a treadmill under comparable conditions. We measured hemodynamic responses over SMA and PFC during 10 walks (each lasting 20 s.) per condition and analyzed the data according to the latest recommendations. In addition, we listed technical issues, stopped the time needed to don fNIRS, and used a questionnaire to assess acceptability.

**Results:** Hemodynamic responses varied largely between participants. Participants with a typical hemodynamic response (i.e., increased oxygenated hemoglobin concentration) showed large cortical activations during walking in Andago compared to treadmill walking (large effect sizes, i.e., for SMA: *r* = 0.91, *n* = 4; for PFC: *r* = 0.62, *n* = 3). Other participants showed atypical (SMA: *n* = 2; PFC: *n* = 4) or inconclusive hemodynamic responses (SMA: *n* = 5; PFC: *n* = 4). The median time for donning fNIRS was 28 min. The questionnaire indicated high acceptance of fNIRS, despite that single participants reported painful sensations.

**Discussion:** Repetitive increased activation of cortical areas like the SMA and PFC might result in long-term neuroplastic changes underlying improved functional outcome. This cross-sectional pilot study provides first numbers on hemodynamic responses in SMA and PFC during walking in Andago in children with neurological impairments, reveals that only a small proportion of the participants shows typical hemodynamic responses, and reports that fNIRS requires considerable time for donning. This information is needed when designing future longitudinal studies to investigate whether increased brain activation of SMA and PFC during walking in Andago could serve as a biomarker to identify potential therapy responders among children and adolescents undergoing neurorehabilitation.

## Introduction

Task-specific neurorehabilitative treatments build on motor learning principles promoting neuroplasticity of brain areas (1), both in adult and pediatric patients. However, predicting the functional outcome after a brain lesion in children (i.e., with a congenital or early acquired lesion) remains very challenging [see also (2, 3)]. On the one hand, studies from the mid-1930s in Macaque monkeys suggest that neuroplasticity in young animals exceeds that of older ones [i.e., referring to the studies of Margaret Kennard, or the “Kennard Principle,” see also (4)]. These studies argue for the greater flexibility of the immature brain with associated good recovery and functional outcome. On the other hand, the “Hebb principle” is based on the theory that neurons that “fire together wire together” and thus form neuronal networks. It argues that the young brain is particularly susceptible to insults during this period, i.e., during early brain development (5). Anderson and colleagues stated that plasticity and vulnerability represent extremes along a “recovery continuum,” where a child's outcome falls along this continuum depending on injury factors (severity, nature, age) and environmental influences (family, sociodemographic factors, interventions) (6). Furthermore, Krageloh-Mann et al. provided evidence that these principles are not mutually exclusive (7).

Children and adolescents and their parents consider improving gait as one of their primary goals (8). During recent years, rehabilitation therapy technologies for the lower extremities have been introduced to the field, complementing conventional therapies like physiotherapy. On the one hand, such technologies could increase at an early stage during rehabilitation the number of task-specific repetitions, which is an essential factor underlying neuroplastic changes and improved functional outcome [see, for example (9)]. On the other hand, such technologies might allow practicing more complex tasks requiring higher cognitive demands earlier in the rehabilitation process, assuming that this would result in a better transfer of improved functional outcome from the safe, standardized therapeutic environment to the more complex and challenging daily life situation. Despite that the evidence for the effectiveness of such technologies is increasing for children with cerebral palsy (CP) (10), and it is likely that also other young patient groups could profit from such interventions, we know little about how we can improve the personalized application of such technologies to facilitate the neuroplastic changes promoting long-term improvement. Besides that the technology should target meaningful goals defined by the young patients and their parents, be well-adjustable to the anthropometrics of the patient, and provide bodyweight unloading and physical support as needed (i.e., as much as needed but as little as possible), we are unaware of any biomarkers that could predict the effectiveness of a particular technology for an individual patient.

In a previous study, we investigated the clinical utility of the rehabilitation therapy device Andago® [Hocoma AG, Volketswil, Switzerland; see (11)] in children and adolescents undergoing rehabilitation of gait. The Andago allows for walking over-ground, while the patient wears a harness to partially unload the bodyweight and prevent falls. Among various aspects, we compared stride-to-stride time and inter-joint kinematics between walking in the Andago vs. walking with the same level of bodyweight support on a conventional treadmill. The results showed that the young patients walked with higher stride-to-stride variability and increased variability in inter-joint kinematics when walking in Andago than during treadmill walking. Interestingly, various patients reported that training in Andago was closer to training in reality and required more concentration than walking on a treadmill (11). This is in line with some of the patients' feedbacks during clinical therapy, where patients navigate over the floors of the rehabilitation center and need to plan and adjust their walking pattern to cope with the environmental demands.

Planning and adjusting walking to changing environmental demands requires more complex cortical control than “just” the primary motor cortex, cerebellum, brainstem, and “central pattern generated spinal stepping” (12). Indeed, various studies have identified cortical areas like the supplementary motor area (SMA), prefrontal cortex (PFC), premotor cortex, primary motor cortex, primary somatosensory cortex, and sensorimotor cortex that are associated with control of human locomotion [for an excellent review see (13)]. For example, while the SMA becomes activated during movement planning (14), and it has been shown that SMA activity is positively correlated with gait variability during forward-walking (15), the PFC becomes activated with increased cognitive-locomotor demands (16). Furthermore, Harada et al. showed that the SMA and the PFC cooperate with other motor-related areas to control gait (17).

Based on the results from (11), feedback from patients, and our clinical observations, we assume that compared to walking on a treadmill, the Andago might indeed require higher activation levels of the SMA and PFC. We assume that children and adolescents with neurological gait impairments who activate these brain areas during a therapy session and would repetitively do so during an intensive rehabilitation program might be able to induce long-term neuroplastic changes in the involved brain regions underlying improved functional outcome. Such a patient could be considered a “responder” to Andago therapy, and the increased activation levels could be a “biomarker” identifying such a responder at onset of the rehabilitation program. Indeed, as part of the ongoing efforts in improving the personalized application of therapy, the search for “biomarkers” that can differentiate between therapy “responders” and “non-responders” is a hot topic. However, even in larger patient groups like adults with stroke, where several interesting biomarker candidates have been identified, only a few are currently available for clinical use (18).

The first aim was to investigate whether we can identify children and adolescents with neurological impairments who show increased activation of the SMA and PFC when walking in Andago vs. walking on a treadmill. We selected functional Near-Infrared Spectroscopy (fNIRS) to assess changes in cortical activity indirectly through the hemodynamic response of the brain (19). In contrast to various other technologies, fNIRS can be used while moving, and it has been validated for motor (i.e., finger tapping) and cognitive tasks using functional magnetic resonance imaging (fMRI) (20).

The second aim was to assess the practicability and acceptability of fNIRS in children when walking. Although the first study that investigated walking-related cortical activations using fNIRS was published already 20 years ago (21), we are unaware of studies investigating the practicability of fNIRS for clinical applications. We consider this particularly relevant for children who might not be as compliant as adults.

Overall, this pilot study should provide first numbers on hemodynamic responses in SMA and PFC during walking in Andago in children with neurological impairments. It should also inform about the practicability and acceptability of fNIRS in these young patients. We consider this information important for designing future longitudinal studies to investigate whether increased brain activation of SMA and PFC during walking in Andago could serve as a biomarker to identify potential therapy responders among children and adolescents undergoing neurorehabilitation.

## Methods

### Participants

We recruited patients of the Swiss Children's Rehab of the University Children's Hospital Zurich in Affoltern am Albis, Switzerland. The inclusion criteria were having a neurological gait impairment, being taller than 125 cm and younger than 18 years old, and understanding and accomplishing simple instructions. The head circumference should be at least 50 cm to fit our smallest fNIRS cap. Participants should be able to walk with dynamic bodyweight unloading for 20 s without stopping and for at least 100 m with pauses. Children or adolescents and their parents should have provided informed consent. Excluded from the study were patients with unconsolidated fractures or bone fragilities of the lower extremities, skin lesions in the harness' area which could not be protected, and impaired head control and inability to maintain an upright position. Furthermore, we did not include children or adolescents who were unable to communicate discomfort or pain. We also excluded those with an implanted baclofen pump causing discomfort.

We characterized the participants by age, bodyweight, height, and diagnosis. We quantified gross motor functioning with the Gross Motor Function Classification system (GMFCS) for children with CP (22). For all children, we quantified the gait performance by the Functional Mobility Scale (FMS), the Gillette Functional Assessment Questionnaire (FAQ), and the Functional Ambulation Categories (FAC). The FMS classifies the functional mobility of children considering the assistive devices needed. The test assesses the assistive devices needed to complete 5, 50, and 500 meters and is rated from 1 (indicating use of a wheelchair) to 6 (independency on all surfaces). The FAQ assesses the functional mobility in different environments. The FAQ provides an ordinal scoring ranging from 1 (the child cannot take any steps) to 10 (the child walks, runs, and climbs on level and uneven terrain without difficulty). The FMS and FAQ have been tested extensively for their psychometric properties (23–25). The FAC is a functional test that evaluates the amount of assistance needed for walking, independently of the use of assistive devices, on a scale from 0 (requires maximum help) to 5 (independent walking) (26).

To obtain an overall “gait score” for gait performance, we calculated for each test (i.e., the FMS, FAQ, and FAC) the percentage of the maximum score reflecting good walking performance, which we then averaged. A child with a 100% gait score would have the best possible score in each test.

### Ethics

Ethical approval was received by the cantonal ethic committee of Zurich (BASEC-Nr. PB_2016-01843). Participants and parents were informed verbally and in writing. Informed consent was provided in accordance with the Declaration of Helsinki. Participants younger than 15 years provided only verbal consent, and participants aged 15 years or more provided both verbal and written consent. Parents signed the consent of the children for all age groups.

### Study Design and Measurement Protocol

**Figure 1:**
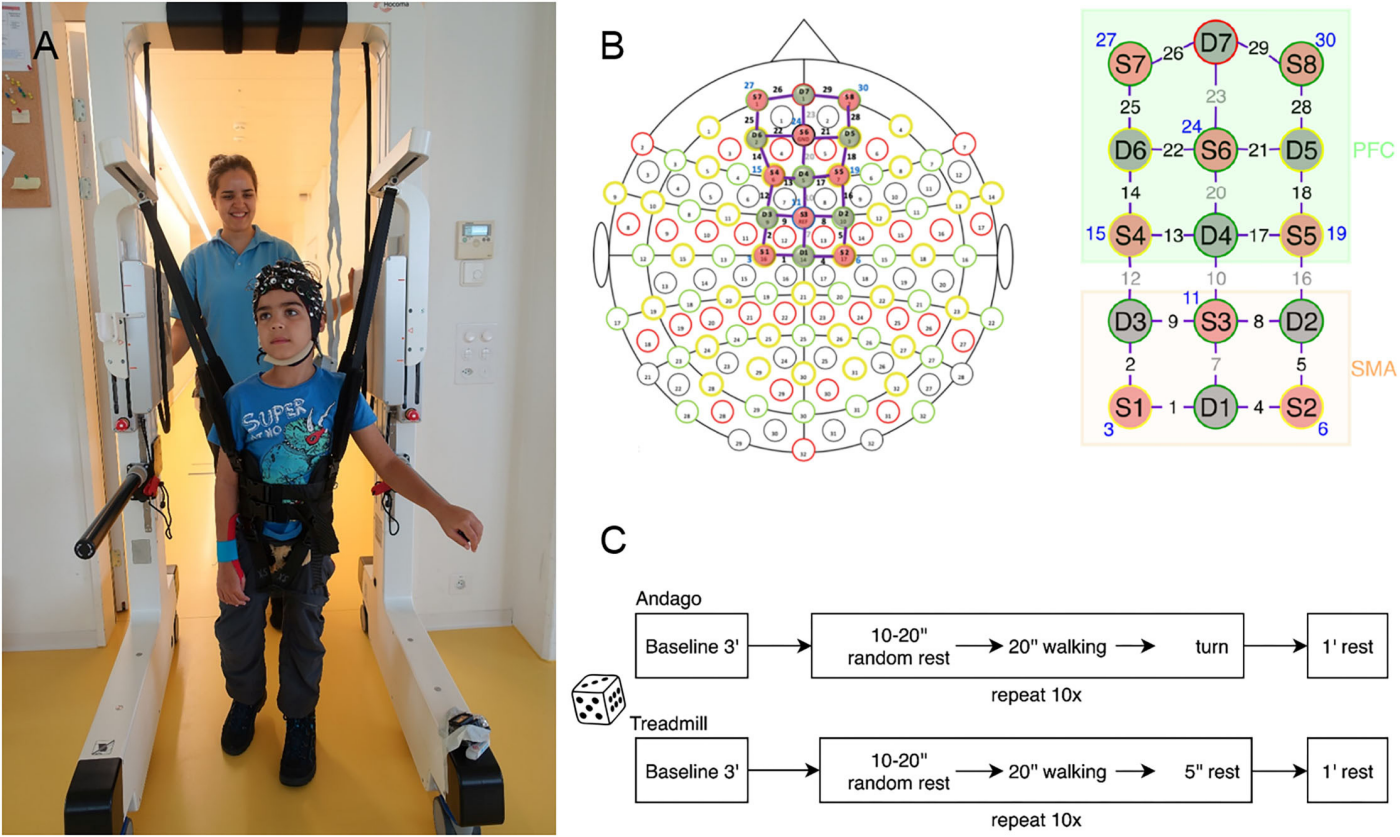
General methodology. **(A)** Young participant walking in the Andago wearing the fNIRS cap. We mounted the Notebook for data-capturing on top of the Andago. **(B)** We configured the optodes to cover the Supplementary Motor Area (SMA) and the Prefrontal Cortex (PFC). Of the 30 channels, eight channels were short channels (displayed in blue). Six channels covered the SMA, 10 channels the PFC. **(C)** Randomization decided whether the participant started with the Andago or the treadmill condition. After the initial baseline measurement, the participants walked 10 times in each condition. To avoid anticipatory effects, they started walking after a rest that varied randomly between 10 and 20 s.

This cross-sectional experimental randomized controlled study included two appointments. During the first appointment, participants could familiarize themselves with walking in the Andago and on the treadmill. During this appointment, we also measured body height, bodyweight, and head circumference. During the second appointment, participants wore the fNIRS cap. They walked in the Andago (Figure 1A) and on the treadmill with similar levels of bodyweight unloading at their preferred walking speed with an upper limit of 3.2 km/h, coinciding with the maximum speed of Andago. In the cap, the optodes were configured to cover the SMA and PFC (Figure 1B). The fNIRS box was stowed with the laptop on the top of the Andago, exempting the children from carrying the equipment in a backpack and allowing them more freedom to move around.

The order of whether a participant started with the Andago or treadmill was randomized using a Matlab (version R2017a, MathWorks, Natick, MA, USA) algorithm. Subsequently, the participants performed 10 consecutive walking trials, each lasting 20 s, at their preferred walking speed (Figure 1C). The trials were preceded by a resting period of random length (between 10 and 20 s) to avoid anticipatory effects (27). After each trial, participants had a minute rest. During the baseline and the rest phases, participants were standing. They had to hold on to the handrails of the devices to prevent cortical activation caused by keeping balance while standing (28), whereas during walking, they were allowed to hold the bars only if necessary. The trials were filmed with a camera to assist in the interpretation of motion artifacts and to document potential adverse events.

### Andago and Treadmill

Andago (V2.0) is a rehabilitation therapy technology for over-ground gait rehabilitation with few spatial limitations. The robot has two detachable handrails, which are helpful for patients with moderate trunk control. Patients are secured with a harness connected to a dynamic weight supporting system, ensuring the safe practice of walking and balance exercises. Bodyweight unloading can be set up to 55 kg while the patient is standing upright. It is dynamic, i.e., it provides a constant bodyweight unloading during the gait cycle. During walking, the therapist can increase or decrease weight support without stopping. In the front, sensors are integrated that detect collisions. The Andago has three different control modes: (i) “Patient-following mode”: the device follows the movements of the patient in any direction. This is possible due to sensors that register the force and direction in which the patient is walking. These sensors are located between the harness and the suspension system. They allow, in combination with the motorized wheels, to follow the patient. Thus, Andago adapts its speed to the patient who can accelerate, decelerate, stop, turn, and even walk backward at any time during the therapy. (ii) “Straight-line mode”: walking is limited to a straight line (backward or forward), and (iii) “Manual mode,” where the therapist can steer the device with a remote controller. In this modus, the sensors become deactivated, and the system does not follow the patient's movements. The system is limited to a maximal speed of 3.2 km/h. This Class IIa device weighs 185 kg and measures 107 cm (front-back) by 195 cm (height) by 85 cm (width; inner width is 67 cm), making it slender enough to pass through standard doors. Between the harness and the device, sensors are mounted that register the force and direction.

While we initially intended to use Andago in the “patient-following mode,” we noticed during initial pilot tests that this was too difficult for several children. Therefore, during this study, the Andago was used in a “straight-line mode.” To turn with the device, the therapist guided the robotic device manually.

Participants also walked on the treadmill that is part of our Lokomat Pro V6 system, which also includes a bodyweight unloading system. Of course, we used the treadmill without the Lokomat and set the same walking speed and bodyweight unloading levels between the Andago and treadmill conditions.

### fNIRS

fNIRS is a neuroimaging technique that measures changes in cortical activity on the principle of neurovascular coupling, i.e., activated brain regions show higher metabolic demands increasing the blood flow to these regions (29). Neural activation causes a hemodynamic response in the activated brain region, observed through a concentration increase of oxygenated hemoglobin (O_2_Hb) and a concentration decrease in deoxygenated hemoglobin (HHb), measured as a function of the absorbed near-infrared light in the underlying cortical tissue.

We used the NIRSport 8x8 system (NIRx Medical Technologies LLC, Berlin, Germany). The device operates at two wavelengths (wl1 = 760 nm, wl2 = 850 nm) with a sampling frequency of 7.81 Hz. We applied a setup of 22 long- and 8 short-distance channels covering the PFC and the SMA (see also Figure 1B). The sources and detectors were arranged on a textile EEG cap (EASYCAP, Herrsching, Germany). We fixed all the cables with wire organizers to provide the best possible stability during movement. Stabilizing distancers were placed between the optode holders to ensure a fixed length between the probes.

The optodes configuration (sources and detectors, see also Figure 1B) was made according to the International 10/20 system for EEG recording with the help of the fNIRS Optode Location Decider (fOLD) toolbox (30). This tool defined channels recording the SMA (orange part, Figure 1B) with 80% specificity and channels covering the PFC (green part, Figure 1B) with 70% specificity. The channels located on the midline of the montage (channels 7, 10, 20, and 23) were excluded from the responder analysis as they covered the fissura longitudinalis cerebri, and fNIRS has limited sensitivity to hemodynamic changes in deep-brain regions (31). Furthermore, channels 12 and 16 were excluded to maintain the specificity in the definition of the two regions of interest. Short-distance detectors were located near the sources and formed short channels with a smaller distance between emitter and detector to penetrate the superficial layers of the scalp only (29). The recorded data from the short separation channels were subtracted from the nearest long-distance channel recording in the cortex to remove the influence of superficial hemodynamics.

To reduce the pressure on the scalp, rubber protections underlying the short-distance detectors were used for the two ventral lines of the montage setup, corresponding to S6, S7, and S8. To fix the optodes to the scalp, we employed spring holders with different grades of pressure to assure more stability with the lowest possible pressure and shorten the preparation phase (17). The company classifies the spring holders as normal- (II), soft- (I), or zero-pressure (0). Before starting device calibration, we adjusted the spring holders according to the participants' preferences. We strived for an optimal contact between the optodes and the scalp while maximizing the comfort of the cap to maintain compliance of the participants during the measurements. In addition, at the onset of the experiment, children were asked if the pressure exerted by the optodes induced painful sensations and if it was possible to wear the cap for more than 1 h with the permission to stop the experiment at any time.

When the optodes and the cap were fixed on the participant's head, the device was calibrated to reach the best possible signal. During the calibration, the gain setting, which quantifies the amplification of the light necessary to obtain a signal, was defined for each channel. Channel gain was defined with a value between 0 and 3, with a 10-fold amplification of the signal between each step. If no signal amplification was necessary, the channel had a gain of 0, whereas weaker light signals needed an amplification to ensure a decreased signal-to-noise ratio. If the light signal amplification was not sufficient to generate a signal that could be detected, the channel was considered “lost.”

### Data Analysis

For the fNIRS data analysis, we used the software nirsLAB (version v2017.06, NIRx Medical Technologies, Glen Head, NY, USA) and Matlab. The consecutive steps are shown in Supplementary Figure 1.

#### Data Quality

Data quality was checked by the software for detector saturation, which was mainly caused by reduced optode-skin contact and resulted in detectors receiving too much light and providing non-reliable information. In case of saturation, intervals with a maximum of four consecutive frames were interpolated, and the signal was restored according to the default settings of nirsLAB (32). The edited attenuation signal was then exported for both wavelengths (wl1 and wl2) and imported into Matlab software for pre-processing operations. Here, data quality was checked once again. If a channel still presented saturation after interpolation, the specific segments were excluded from further analysis. Additionally, the signal-to-noise ratio of the data was quantified through the coefficient of variation (CV) within the channels and within the single segments.


(1) $$\begin{array}{l} Coefficient\;of\;variation\;\left(CV\right)=SD/Average \end{array}$$

The CV was calculated for each channel and for its segments for both wl1 and wl2. Since fNIRS signal fluctuations over time are usually small in comparison to the average signal, the CV is generally low. We excluded all channels with an overall CV exceeding 15% during the measurement. All 10 segments of each walking trial were then investigated individually. If a segment had a CV that exceeded 5% for one of the two wavelengths, it was excluded from further analysis (33). Since the recorded signal from the long-distance channels was corrected based on the nearest short-distance channel, excluding a short-distance channel also resulted in the exclusion of the corresponding long-distance channels. Finally, we excluded the channels with a gain exceeding 2 because signal quality is considered excellent only for gains between 0 and 2 (32). In summary, only the channels with a CV smaller than 15% and a gain not exceeding 2, as well as the segments from valid channels without detector saturation and with a CV smaller than 5% were kept for the next steps.

In nirsLAB, the data were then converted to concentration changes of O_2_Hb and HHb with the modified Beer-Lambert law for scattering media (34). In this step, an age-dependent differential path-length factor (35) and the measured distance between source 1 and detector 1 (mean 3.2 cm) were employed.

#### Motion Artifact Removal

The hemodynamic data were imported in Matlab to correct for motion artifacts using the Motion Artifact Removal Algorithm (MARA) (36). This step was performed for each participant, channel, and O_2_Hb and HHb separately, observing the moving standard deviation of the data and manually setting a threshold for the definition of the artifacts. Motion artifacts of both walking sessions (Andago and treadmill) were corrected together to facilitate the comparison. Peaks above the threshold were considered artifacts and corrected using the algorithm that denoised the problematic segments by subtracting the spline interpolation from the signal of this segment (36). Approximately 700 plots were checked independently by a second investigator, and the divergent opinions for the thresholds were compared to reach optimal data quality. All the peaks in the stimulation period counted as excluded peaks and were automatically registered with the thresholds and the corresponding signal-to-artifact ratio (SAR) for each subject with the corresponding channels.

#### Segmentation, Filtering, and Block Averaging

In the following step, the corrected hemodynamic data were segmented for each channel to isolate the 10 trials performed. The segments were 30 s long subdivided in 20 s walking, 5 s pre-stimulus, and 5 s post-stimulus. To preserve only the information about the cortical activity contained in the signal, a band-pass filter with the Savitzky-Golay filter was applied (37). Subsequently, data were detrended using linear regression and normalized by subtracting the median value of the 5 s pre-stimulus from each segment. To remove superficial interference (like scalp blood flow), data obtained from the long-distance channels were further corrected through a short channel regression, according to Saager and Berger (38). We subtracted the weighted signal from a short-distance channel from the signal of the paired long-distance channel. The vector containing the stimulation indexes for each channel was then accordingly modified. Thus, the block average of all remaining segments was calculated for each valid channel of each participant.

#### Responder Analysis and Subgroups

A typical hemodynamic response following neuronal activation is characterized by an increased concentration of O_2_Hb and a decreased concentration of HHb. For each participant and each region (SMA and PFC), we calculated the median hemodynamic response for each condition (i.e., Andago and treadmill). Theoretically, these median relative concentration changes in O2Hb could have been calculated over up to 6 channels x 10 trials for SMA and up to 10 channels × 10 trials for PFC (see Figure 1B for the number of channels per SMA or PFC) for each condition. However, only those hemodynamic responses that had passed the quality checks previously described were included.

In line with a previous study that found large intersubject variability in hemodynamic responses in neonates (39), we grouped the participants into one of three subgroups. Subgrouping was based on the median hemodynamic responses obtained during walking in Andago. The median trajectory of the relative concentration change in O_2_Hb consisted of 157 data points for each participant and brain region during the stimulus period. For each participant, we calculated the following effect size (ES):


(2) $$\begin{array}{l} ES_{Andago}=Average/SD \end{array}$$

Where “Average” is the average value of the 157 data points and “SD” their standard deviation. The magnitude of the effect size *ES*_*Andago*_ is interpreted as 0.2 small, 0.5 medium, and 0.8 large.

We defined the following subgroups:

■ Group 1 was characterized by a medium-sized *increase* in O_2_Hb during the stimulation period and defined with *ES*_*Andago*_ ≥ 0.5.
■ Group 2 was characterized by a medium-sized *decrease* in O_2_Hb during the stimulation period and defined with *ES*_*Andago*_ ≤ −0.5.
■ Group 3 showed an *inconclusive* response (−0.5 < *ES*_*Andago*_ < 0.5).

When each participant was categorized into one of the three groups, we compared the hemodynamic responses between the Andago and treadmill conditions (separately for the SMA and PFC). As the number of participants per group was small, we applied a non-parametric effect size *r* to interpret the group differences between the conditions:


(3) $$\begin{array}{l} r=\frac{z-value}{\sqrt{N}} \end{array}$$

Where the z-value was derived from the non-parametric Wilcoxon signed rank-test and *N* was the number of observations. We interpreted the magnitude of the effect size *r* as 0.1 small, 0.3 medium, and 0.5 large. Please note that we only performed these analyses for groups 1 and 2 because participants belonging to group 3 had not shown any relevant change in cortical activity during walking in the Andago.

### Practicability

For the practicability part, we investigated the robustness of the technology by noting any technical issues (for example, concerning the connection of the cables, the duration of the battery, breakdowns of the system). Different experiments can have different durations. Therefore, we focused merely on the preparation time for donning the fNIRS technology. This included the time needed to accurately put on the cap, including measuring the head and clearing the hair at optode locations, the time to calibrate the NIRS device, and the time for instructions and preparation.

### Acceptability

The comfort of the fNIRS cap was investigated through a multilevel questionnaire developed and adapted from a questionnaire for wearable computers comfort (40). It enabled assessing different aspects regarding comfort and was rated with a continuous scale from 0 to 10, with 0 meaning comfort and 10 meaning maximal discomfort. The items reflected emotion, attachment, harm, perceived change, movement, and anxiety. The children could read the questions and complete the questionnaire by themselves or with the help of the investigator, who read out the questions. Children received explications if they did not understand the questions and were provided with an example for the extreme values of each item. The questionnaires were filled out at the half and end of the measurement.

## Results

### Participants

Two participants (ID 10 and 14) dropped out after the first session; one had poor compliance, while the other did not have the leg orthosis on hand to walk during the measurement. The characteristics of the 13 remaining participants are listed in Table 1. The data of two participants (ID 6 and 9) was of such poor quality (see also Supplementary Figures 2–5) that we could not include them in the fNIRS analyses (Table 1).

**Table 1:** Characteristics of the participants.

**ID**	**Sex**	**Age**	**Order**	**BW (kg)**	**BH (cm)**	**Diagnosis**	**GMFCS**	**FMS 5**	**FMS 50**	**FMS 500**	**GFAQ**	**FAC**	**Gait score**
1	M	13.9	T	78	178	TBI		5	5	5	9	5	91
2	M	13.5	A	46	149	CP/Post-op	III	2	1	1	4	3	41
3	M	9.2	A	18	125	CP/Post-op	III	2	2	1	6	4	56
4	F	15.4	A	51	155	DD		6	6	6	9	5	97
5	M	8.5	A	56	138	TBI		6	6	6	10	5	100
6	M	12.6	A	47	150	CP/Post-op	III	1	1	1	2	2	26
7	F	11.6	T	50	140	CP	IV	2	1	1	4	2	34
8	M	15.7	T	54	181	Stroke		6	6	5	9	5	95
9	M	9.1	T	35	134	CP	IV	2	2	1	6	4	56
11	M	13.8	T	42	160	CP/Post-op	III	3	2	1	4	3	44
12	M	8	A	25	125	Hemiparesis		1	1	1	3	3	36
13	M	14.4	A	73	182	Demyelination		4	4	2	8	4	72
15	M	12.3	T	33	144	CP/Post-op	III	3	3	2	6	2	48
**Median**		**12.6**		**47**	**149**			**3**	**2**	**1**	**6**	**4**	**56**
**IQR**		**9.2–14.2**		**34–55**	**136–169**			**2–5.5**	**1–5.5**	**1–5**	**4–9**	**2.5–5**	**38.5–93**

*M, male; F, female; T, participant walked first on the treadmill; A, participant walked first in Andago; BW, bodyweight; BH, body height; TBI, traumatic brain injury; CP, cerebral palsy; Post-op, after neuro-orthopedic surgery; DD, developmental disorder; GMFCS, Gross Motor Function Classification System; FMS, Functional Mobility Scale; GFAQ, Gillette Functional Assessment Questionnaire, walking scale; FAC, Functional Ambulation Categories. NIRS data quality of ID6 and ID9 was poor. Data of these participants was only used for practicability and acceptability*.

### Identifying Participants With Increased Activation of SMA and/or PFC

The O_2_Hb concentration changes of each participant for each condition and cortical area are shown in Table 2. We grouped the participants according to the *ES*_*Andago*_ (see also Responder analysis and subgroups), which quantified the hemodynamic responses obtained during walking in Andago. We grouped once for the SMA and once for the PFC. Group 1 includes those participants who showed a typical response (i.e., an increase in O_2_Hb during walking in Andago). Group 2 included participants who showed an inverse response (i.e., decreased O_2_Hb during walking in Andago). Finally, participants in group 3 showed an inconclusive response.

**Table 2:** Changes in oxygenated hemoglobin levels for each participant.

**Cortical area**	**Group**	**ID**	**Mean** **±** **SD concentration changes of O**_**2**_**Hb (10**^**−4**^**mM)**		***ES_***Andago***_***	**Gait score**
			**Andago**	**Treadmill**		
SMA	1	3	1.48± 1.39	0.94 ± 0.04	1.07	56
		7	1.88± 2.25	0.85 ± 5.8	0.84	34
		13	2.17± 4.18	0.76 ± 0.35	4.12	72
		15	1.46± 1.35	0.71 ± 0.94	1.08	48
	2	4	−1.17± 1.05	−0.03 ± 1.42	−1.11	97
		5	−1.29± 1.78	1.05 ± 1.46	−0.72	100
	3	1	−0.18± 2.17	1.45 ± 2.52	0.08	91
		2	0.04± 0.49	3.25 ± 2.02	0.08	41
		8	−0.65± 3.59	−0.39 ± 0.89	−0.18	95
		11	1.17± 4.18	−3.51 ± 2.27	0.28	44
		12	0.10± 0.80	1.00 ± 2.03	0.12	36
PFC	1	7	1.97± 2.67	2.63 ± 3.96	0.74	34
		13	0.35± 0.55	0.32 ± 0.25	0.65	72
		15	1.02± 0.66	0.27 ± 0.57	1.53	48
	2	1	−0.80± 1.61	0.75 ± 1.09	−0.50	91
		3	−1.12± 2.09	−0.98 ± 3.16	−0.53	56
		4	−1.39± 2.70	−0.64 ± 1.07	−0.51	97
		11	−1.46± 1.84	−1.21 ± 2.47	−0.79	44
	3	2	1.42± 5.42	1.02 ± 5.25	0.26	41
		5	−0.19± 1.73	0.11 ± 4.17	−0.11	100
		8	0.59± 2.72	−0.60 ± 1.17	0.22	95
		12	0.37± 0.97	0.00 ± 2.14	0.38	36

*SMA, supplementary motor area; PFC, prefrontal cortex; SD, standard deviation; O_2_Hb, oxygenated hemoglobin; ES_Andago_, effect size calculated for the Andago condition*.

**Figure 2:**
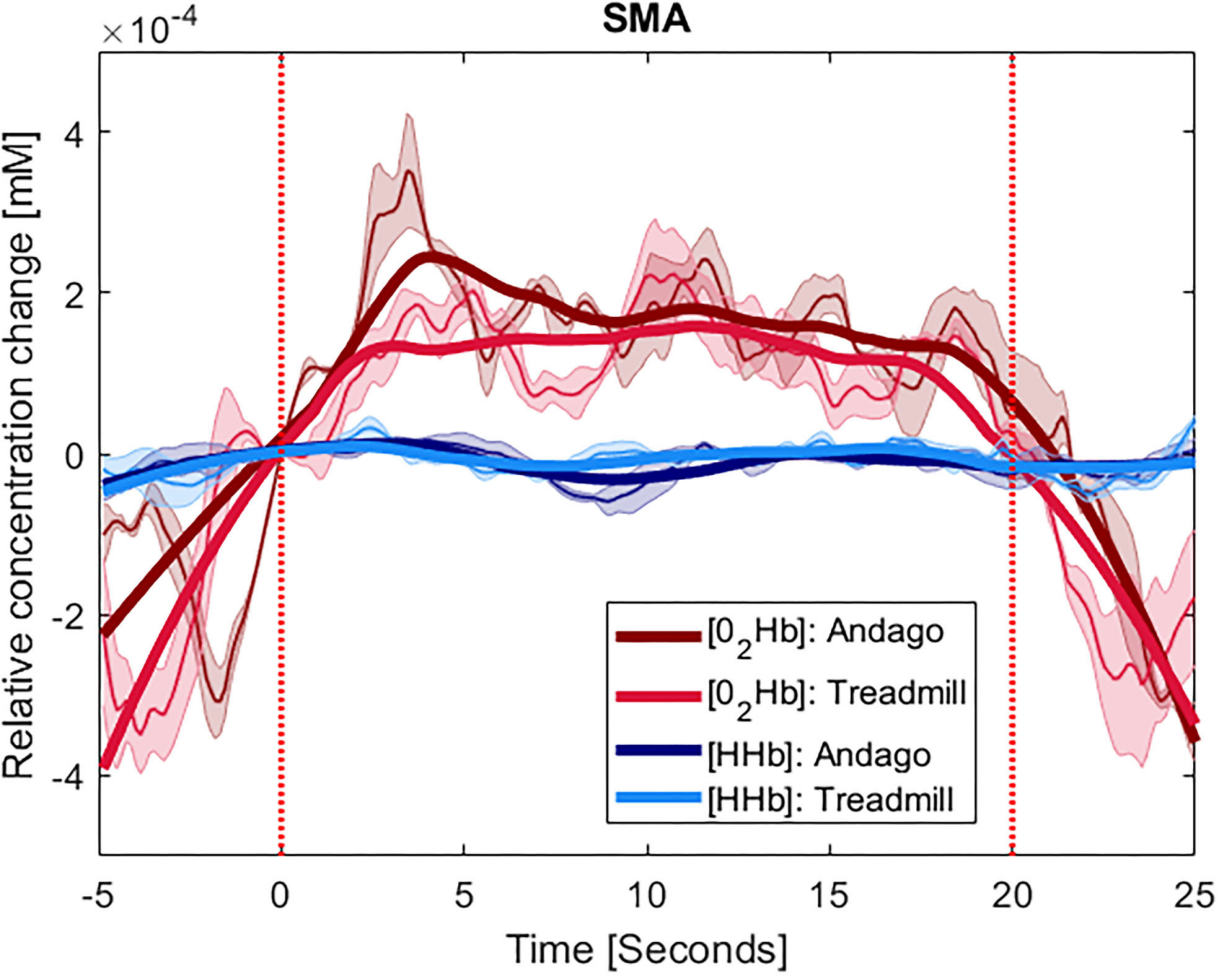
Averaged time-series for group 1 for SMA. The y-axis shows the concentration changes in millimoles per liter (mM) of O_2_Hb in red and HHb in blue, averaged across the participants with at least one valid channel for the area. Thinner lines represent the averaged curves. The shaded area represents the standard error of the mean. The thick lines represent smoothened concentration changes. The x-axis represents the time of the stimulus (20 s) plus the 5 s before and 5 s after it. The vertical red lines indicate the onset and end of the stimulus. The plots displaying the hemodynamic responses for the SMA were averaged over 4 participants (ID 3, 7, 13, and 15).

**Figure 3:**
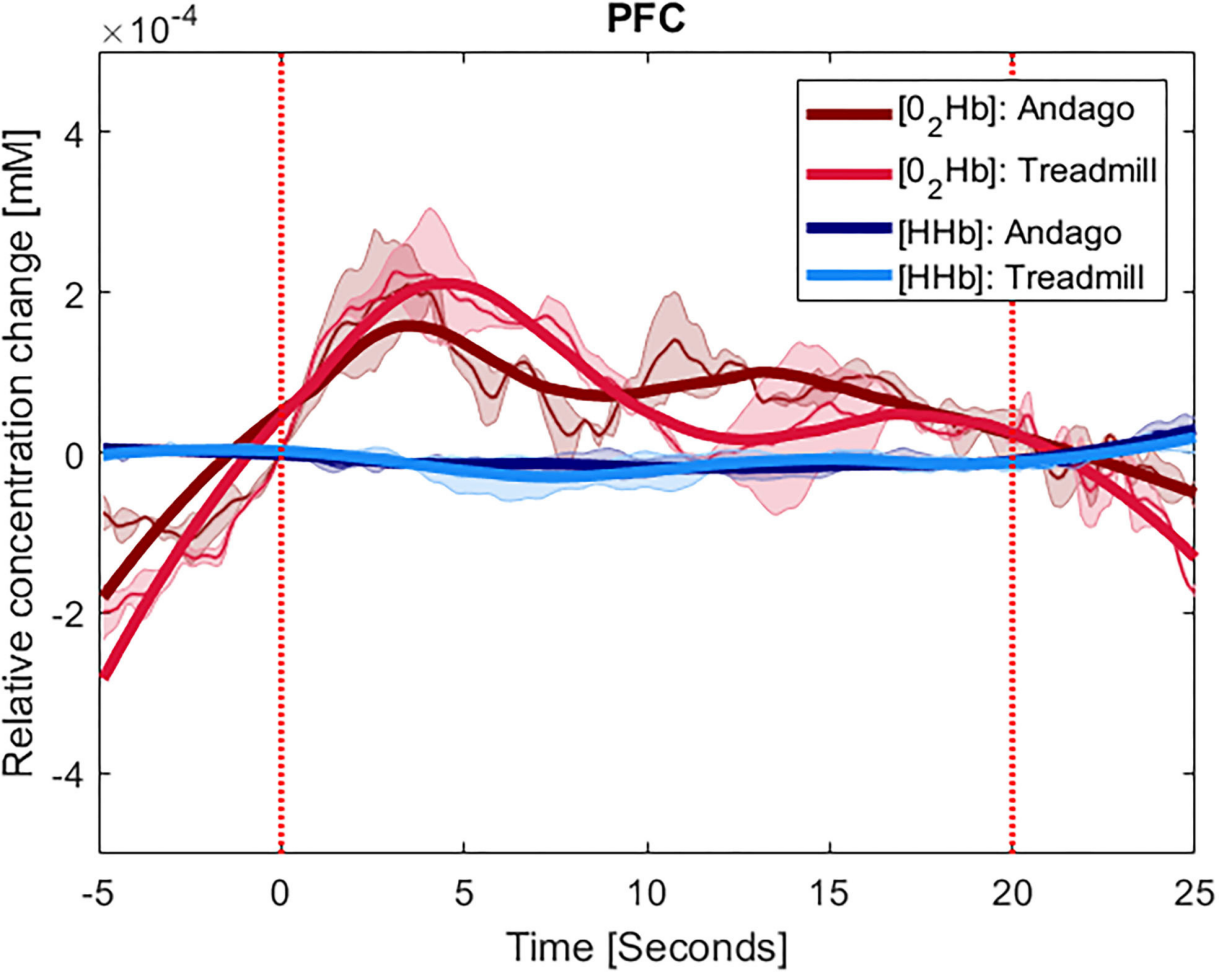
Averaged time-series for group 1 for PFC. The y-axis shows the concentration changes in millimoles per liter (mM) of O_2_Hb in red and HHb in blue, averaged across the participants with at least one valid channel for the area. Thinner lines represent the averaged curves. The shaded area represents the standard error of the mean. The thick lines represent smoothened concentration changes. The x-axis represents the time of the stimulus (20 s) plus the 5 s before and 5 s after it. The vertical red lines indicate the onset and end of the stimulus. The plots displaying the hemodynamic responses in the PFC were averaged over 3 participants (ID 7, 13, and 15).

The plots displaying the averaged hemodynamic responses for O_2_Hb (in red) and HHb (in blue) for the two regions SMA and PFC and the two conditions along with shaded error bars representing the standard error of the mean are shown for group 1 in Figure 2 (SMA) and Figure 3 (PFC), see also Supplementary Figure 6. When comparing the changes in oxygenated hemoglobin concentrations between the Andago and treadmill for the SMA, we found a large effect size *r* of 0.91. The Wilcoxon signed-rank test showed a statistical tendency toward a larger response during walking in the Andago compared to treadmill walking (Wilcoxon signed-rank test, z = −1.826, *p* = 0.068).

For the PFC, we also found a large effect size for the difference in hemodynamic responses between the two conditions (*r* = 0.62). Yet, due to the low number of participants (*n* = 3), the Wilcoxon signed-rank test was not significant (z = −1.069, *p* = 0.29).

The averaged time-series for group 2 (who had shown a relevant decrease in O_2_HB during walking in the Andago) are displayed in Supplementary Figure 7. We did not evaluate the group data for the SMA, as there were only two participants in this group. For the PFC, the effect size *r* was 0 and the *p*-value 1.0.

The averaged time-series for group 3 are shown in Supplementary Figure 8.

When comparing the gait score between the three groups for the SMA, the Kruskal-Wallis test indicated a tendency for a higher gait score for group 2 (Kruskal-Wallis H = 4.541, DF = 2, *p* = 0.10). We did not observe a tendency for a difference in gait score between the three groups for the PFC (Kruskal-Wallis H = 1.144, DF = 2, *p* = 0.56).

### Practicability

We did not experience considerable technical issues with the fNIRS system. The median time needed to put on the cap amounted to 12.1 min and ranged between 7.4 and 26.8 min for the thirteen participants. The median duration for calibrating the fNIRS system was 9.2 min (range: 1.5–21.4 min), and the median time for instructing and preparing the child lasted 8.6 min (range: 5.6–14.3 min). So, the median overall preparation time amounted to 28 min with an IQR of 24 to 37 min and a range between 17.2 and 56.8 min.

### Acceptability

Generally, the questions received low scores indicating high acceptability. However, some participants provided scores up to 10, indicating being nervous or feeling strange when wearing fNIRS technology. The highest score (i.e., lowest acceptability) was observed for the question of whether it felt strange wearing the device, but the median value became better at the end of the study. While the median score for pain was 0 halfway and 0.5 at the end, individual participants reported values up to 6. The responses to all questions are shown in Table 3.

**Table 3:** Acceptability of wearing the fNIRS system.

**Question**	**Halfway**			**End**			***p*-value**
	**M**	**IQR**	**Range**	**M**	**IQR**	**Range**	
How do you feel wearing the device (relaxed/very nervous)?	1.5	0–5	0–10	0.5	0–2.5	0–10	0.11
Do you feel that the device is moving on your head (very stable/it moves a lot)?	0.5	0–1	0–5	0	0–1	0–3.5	0.67
Is the device painful to wear (no pain/much pain)?	0	0–4	0–6	0.5	0–3.5	0–6	0.67
Do you feel strange wearing the device (normal/very strange)?	4.5	1.5–8	0–10	3	0.5–5	0–10	0.049
Does the device affect the way you move (normal/very restricted)?	0	0–2	0–6.5	0	0–1.75	0–5.5	0.46
Do you feel unsecure wearing the device (secure/not at all)?	0	0–3	0–5	0	0–1.25	0–5	0.20

*M, median; IQR, interquartile range; p-value, p-value derived from Wilcoxon signed rank test. Please note that low values indicate comfort*.

## Discussion

We are generally interested in identifying biomarkers for the personalized application of rehabilitation therapy technologies such as the Andago. One candidate biomarker could be increased hemodynamic responses in relevant cortical areas recorded with fNIRS during technology-assisted walking in the target group. For a well-designed longitudinal study investigating the predictive capacity of hemodynamic responses assessed at onset of rehabilitation with functional outcome at the end of therapy in the target group, information on the number and size of the hemodynamic responses is needed to perform a sample size calculation. Furthermore, information on the practicability and acceptability of the fNIRS technology is required to estimate the feasibility of such a trial.

Therefore, the first aim of this pilot study was to investigate whether we could identify children and adolescents with neurological gait impairments who showed increased activation of the SMA and PFC when walking in Andago compared to walking on a treadmill. The second aim was to assess the practicability and acceptability of fNIRS in children when walking with such a technology.

The main results were the following. (i) By using fNIRS, we could identify children with increased hemodynamic responses in SMA or PFC when walking in Andago. However, the inter-individual variability in the responses was large, and only a small proportion of the children could be categorized into this group. (ii) It takes time to don the fNIRS technology in children and adolescents with neurological impairments. Before starting the actual measurement, we needed about half an hour (in some children, it took almost an hour) to prepare the child. Generally, the children accepted the fNIRS technology well, but some felt strange when wearing the technology, and some reported painful sensations.

### Increased Activation of SMA and/or PFC When Walking in Andago

Our first main finding was the large inter-individual variability of hemodynamic responses and the relatively small number of children that showed a typical hemodynamic response in the fNIRS signal when walking in Andago. This might seem a surprise because in an earlier fNIRS study, the authors reported increased activation in the sensorimotor cortices and superior parietal lobule during gait in children with bilateral spastic CP compared to typically developing children (41). A similar finding was reported in a recent electroencephalography (EEG) study where children with CP showed an increased cortical activation during treadmill walking compared to typically developing peers (42). In another fNIRS study, children with bilateral CP showed higher cortical activation than children with unilateral CP, who showed higher activity than typically developing children when performing a distal (i.e., ankle dorsiflexion) task (43). The authors also reported that children with GMFCS level III showed higher activations than those with GMFCS I or II. Apparently, children with gait impairments, for example due to CP, require higher cortical activation levels to control gait than typically developing children. This finding has also been confirmed for other adult patient groups. For example, when comparing activation in the PFC using fNIRS in young and elderly adults and patients after stroke during regular walking and performing more complex walking-related tasks, the highest activations were found for the patients with stroke, followed by the elderly adults and, finally, the young adults (44). The authors' interpretation of their findings was that patients after stroke and elderly adults require increased levels of prefrontal/executive control resources during walking (particularly during more complex tasks).

Nevertheless, from the initially included fifteen participants, we could identify only four who showed an increase in SMA and three who showed an increase in PFC activity (i.e., group 1). While a longitudinal trial investigating the predictive capacity of hemodynamic responses in SMA and/or PFC is needed to verify whether participants showing typical hemodynamic responses during Andago therapy would improve more in functional outcome than those with atypical or inconclusive hemodynamic responses, the small proportion of children showing these typical hemodynamic responses will influence sample size calculations considerably.

The results seemed somewhat stronger for the SMA compared to the PFC. The different number of participants could be one of the reasons why SMA results showed a larger effect size and a tendency for significant difference between the Andago and treadmill condition compared to the PFC. Based on the findings from our previous study showing that walking in Andago results in a higher stride-to-stride variability and increased variability in inter-joint kinematics (11), we can confirm the results from Kurz et al. (15). They found that SMA activity is increased when walking with higher step-to-step variability. Besides the smaller sample, another reason why we observed less clear findings for the PFC could have been because of our decision to use Andago in the “straight-line-mode.” Walking in this mode reduces the need to plan and navigate through the environment. As a consequence, the cognitive demands might have been lower. We expect that walking in the “patient-following-mode” might have resulted in larger activation of PFC. Another reason why the cortical activation did not differ clearly between the two tasks might be the relatively small contrast between the conditions. For example, while some studies investigated differences in cortical activation between patients and healthy participants or between rest and walking or standing and walking [see also (13)], we compared walking on a treadmill vs. walking in Andago at the same speed and with the same level of bodyweight support. Finally, several (particularly older) fNIRS studies might have investigated cortical activation during walking without the use of, for example, short channels in optode arrangements to control for changes in superficial scalp circulation (45). Particularly in those studies that compared tasks with different levels of activation (e.g., rest vs. walking or stance vs. walking), differences in superficial hemodynamics might have influenced the results.

We found several participants who showed a negative hemodynamic response. Such an inverted hemodynamic response has also been reported by Arimitsu et al. (46). They investigated hemodynamic responses to speech in 80 preterm and term neonates and noticed that the atypical response frequently occurred in preterm infants before 36 weeks of postmenstrual age. The authors could only speculate about the underlying physiological mechanisms. They suggested that it might be explained by immature neurovascular and/or metabolic systems in the developing cortex. Specifically, immature functioning of the synaptic structure with less myelination might result in inefficient energy use requiring more oxy-hemoglobin. Because of the immaturity of the arteriole vessels and capillaries, blood flow may not be sufficient in response to brain activity (46). Karen et al. also noticed in neonates a high intersubject variability in hemodynamic responses to visual stimulation (39). Based on the hemodynamic responses (positive, negative, and inconclusive hemodynamic response), they grouped the neonates (i.e., similar as we did). They found that the characteristics correlated with differences in the weight of the neonate at measurement.

We can only speculate why we found a large proportion of the participating children and adolescents with an atypical or inconclusive hemodynamic response. One reason could be that considering the limited attention span of children, atypical hemodynamic responses due to distracting factors can be expected during long-lasting sessions. Furthermore, the walking ability could have influenced cortical activation. While we noticed that the participants who showed a negative response in the SMA were those with the best gait score (was walking in Andago too easy for them?), we could not confirm this for the participants showing the negative response in the PFC. Unfortunately, due to the small number of participants in each subgroup, it is too early to draw any conclusions on the relationship between walking ability and type of hemodynamic response when walking in the Andago. Also, it is known that walking speed influences cortical activation [for an overview, see (13)]. In our study, the participants walked at their preferred walking speed, meaning that four participants walked at a speeds below 1 km/h, four participants between 1 and 2.1 km/h, and five participants walked between 2.1 and 3.2 km/h. Additionally, while all participants had a neurological diagnosis and required neurorehabilitation to improve gait, it was a heterogeneous patient group in terms of the time of lesion (e.g., congenital vs. later acquired), the time after the acquired lesion, the location and size of the lesion, etc. Further research is needed to clarify why these participants showed atypical or inconclusive cortical activations during this task.

### Practicability and Acceptability

A study from 2017 investigating the application of fNIRS in patients with gait disorders suggested that the translation of fNIRS in clinical practice should be encouraged, either for assessing the effectiveness of rehabilitation treatments or for evaluating neuro-functional correlates of gait disorders in ecologically valid conditions (47). Based on the significant inter-individual hemodynamic responses, but also on our practicability and acceptability results (for example, the long duration needed for the preparation, the need for highly trained personnel, and the time required for data processing), we consider it too early to transfer such technology to clinical practice, particularly in the pediatric field. To overcome practical barriers, preparation times should be reduced. One development could be more uncomplicated techniques to comb the hair away from the optodes, which could help in cases where the effect of the spring-holders is not sufficient to reach an excellent optode-scalp contact. Some participants reported moderate pain due to the pressure exerted by the optodes. While such a pressure might be tolerable for 10–20 min, it isn't easy to sustain for longer sessions. In our study, the participants wore the cap for up to almost 90 min resulting in pressure marks, particularly on hairless regions of the head. For children, it might be beneficial to use dual-tip optodes or optodes with a flatter tip similar to those used in infants to improve comfort and tolerate longer-lasting fNIRS measurements.

### Methodological Limitations

This study has several limitations. First, the number of participants in this pilot study was low. Due to the high intersubject variability in hemodynamic responses, we had to divide the participants in three subgroups, precluding serious statistical analyses. However, we suggest that these results are valuable for future studies. The results presented in Table 2 can be used to perform sample size calculations. An important lesson learned from this pilot trial is that only 4/11 participants (i.e., 36%) showed a typical hemodynamic response in SMA and only 3/11 or 27% in PFC. It should be noted that the participants of this pilot study were not randomly selected among all patients undergoing Andago training, which could have affected the exact proportions. Still, when planning future studies investigating hemodynamic responses in a comparable patient group, researchers should include in their sample size calculations that only a (small) proportion of the participants might show typical hemodynamic responses.

In addition, the group was heterogeneous, and we did not investigate the exact lesion size and location. Furthermore, fNIRS technology cannot be applied to each individual reliably. ID 6 had dense hair, and the contact between optodes and the scalp was difficult from the onset on. ID 9 had dark skin and hair pigmentation, and many channels were already lost during calibration due to the high concentration of melanin. Data quality may also have been affected by anatomical variations in the thickness of the skull and the shape of the head that could have resulted in a less-optimal contact of certain optodes. Nevertheless, the rates of excluded channels were overall satisfying, considering that participants with a “good” calibration had a maximum of 5 channels excluded out of the 30 measured. Moreover, on average, almost 5 out of 20 trials contained a motion artifact. If an artifact occurred during the stimulus (i.e., Andago or treadmill walking), this often led to the exclusion of the channel due to a high CV. All the remaining segments and channels were corrected with the MARA but the correction of an artifact within the stimulation time remains critical also using this algorithm. While we showed a learning curve in preparation time, and more experienced researchers might have needed less time, it still requires high expertise, making the transfer to clinical application challenging.

## Conclusions

This study should be considered a pilot study investigating hemodynamic responses in SMA and PFC when walking in the Andago. While we could identify individual patients who showed increased SMA or PFC activation when walking in Andago, we noticed a large intersubject variability in responses. It remains to be confirmed that young patients showing a typical hemodynamic response in SMA or PFC would indeed be “responders” when participating in intensive long-term training with Andago, showing superior neuroplastic reorganization and improved functional outcome compared to patients lacking such activations. In other words, we need further in-depth studies to learn whether the typical hemodynamic response pattern that we observed in group 1 could be used as a potential therapy-device-specific “biomarker.” In addition, despite that the fNIRS technology was generally well-accepted by the young participants, some practical and acceptability issues need to be addressed. This makes it currently difficult to implement such measurements as a clinical routine to quantify treatment effects and neuroplastic changes.

## Data Availability Statement

The original contributions presented in the study are included in the article/Supplementary Material, further inquiries can be directed to the corresponding author/s.

## Ethics Statement

The studies involving human participants were reviewed and approved by Cantonal Ethical Committee of Zurich. Written informed consent to participate in this study was provided by the participants' legal guardian/next of kin. Written informed consent was obtained from the minor(s)' legal guardian/next of kin for the publication of any potentially identifiable images or data included in this article.

## Author Contributions

HH was involved in the study design, wrote parts of the manuscript, interpreted the results, supervised the project, provided funding, and obtained the ethical approval. AB was involved in the study design, performed the measurements, analyzed the data, wrote parts of the manuscript, and interpreted the results. AG was involved in the study design, performed the measurements, analyzed the data, interpreted the results, and supervised AB. All authors critically reviewed the manuscript and agreed with the final version.

## Funding

This work was funded by the Anna-Müller Grocholski Foundation. HH was paid by a grant from the Mäxi Foundation. The Foundations were not involved in any part of the study.

## Conflict of Interest

The authors declare that the research was conducted in the absence of any commercial or financial relationships that could be construed as a potential conflict of interest.

## Publisher's Note

All claims expressed in this article are solely those of the authors and do not necessarily represent those of their affiliated organizations, or those of the publisher, the editors and the reviewers. Any product that may be evaluated in this article, or claim that may be made by its manufacturer, is not guaranteed or endorsed by the publisher.

## Abbreviations

CP: Cerebral Palsy
ES_Andago_: Effect Size (calculated for Andago condition)
FAC: Functional Ambulation Categories
FAQ: Gillette Functional Assessment Questionnaire, waking scale
FMS: Functional Mobility Scale
fNIRS: functional Near Infrared Spectroscopy
GMFCS: Gross Motor Function Classification System
HHb: deoxygenated hemoglobin
IQR: Interquartile Range
O_2_Hb: oxygenated hemoglobin
PFC: Prefrontal Cortex
SMA: Supplementary Motor Area.
