# Supplementary material for: Prefrontal Cortex and Supplementary Motor Area Activation During Robot-Assisted Weight-Supported Over-Ground Walking in Young Neurological Patients: A Pilot fNIRS Study
Source: Front Rehabil Sci. 2021 Dec 10;2:788087. doi: 10.3389/fresc.2021.788087 (PMC9397849; doi:10.3389/fresc.2021.788087)
Supplement: Supplementary file 1 [file Data_Sheet_1.docx]

Supplementary Material


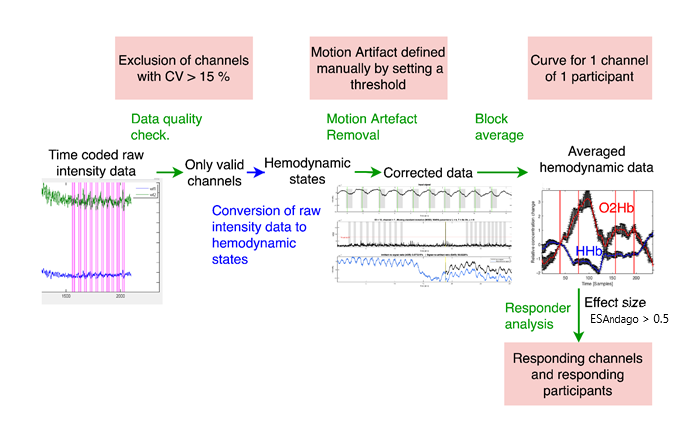


**Supplementary Figure 1.** **fNIRS Data pre- and post-processing.**

The raw intensity data informs about the behavior of the two wavelengths (wl1 blue, wl2 green) during the whole experiment. The data were subsequently time-coded (purple bands represent the stimulation times) and cutted. In the pre-processing step, the coefficient of variation (CV) of both wavelengths was checked and subsequently in the post-processing steps the movement artifacts were corrected (MARA). Attenuation data of wl1 and wl2 were then converted and averaged to obtain hemodynamic changes of O_2_Hb (red) and HHb (blue).


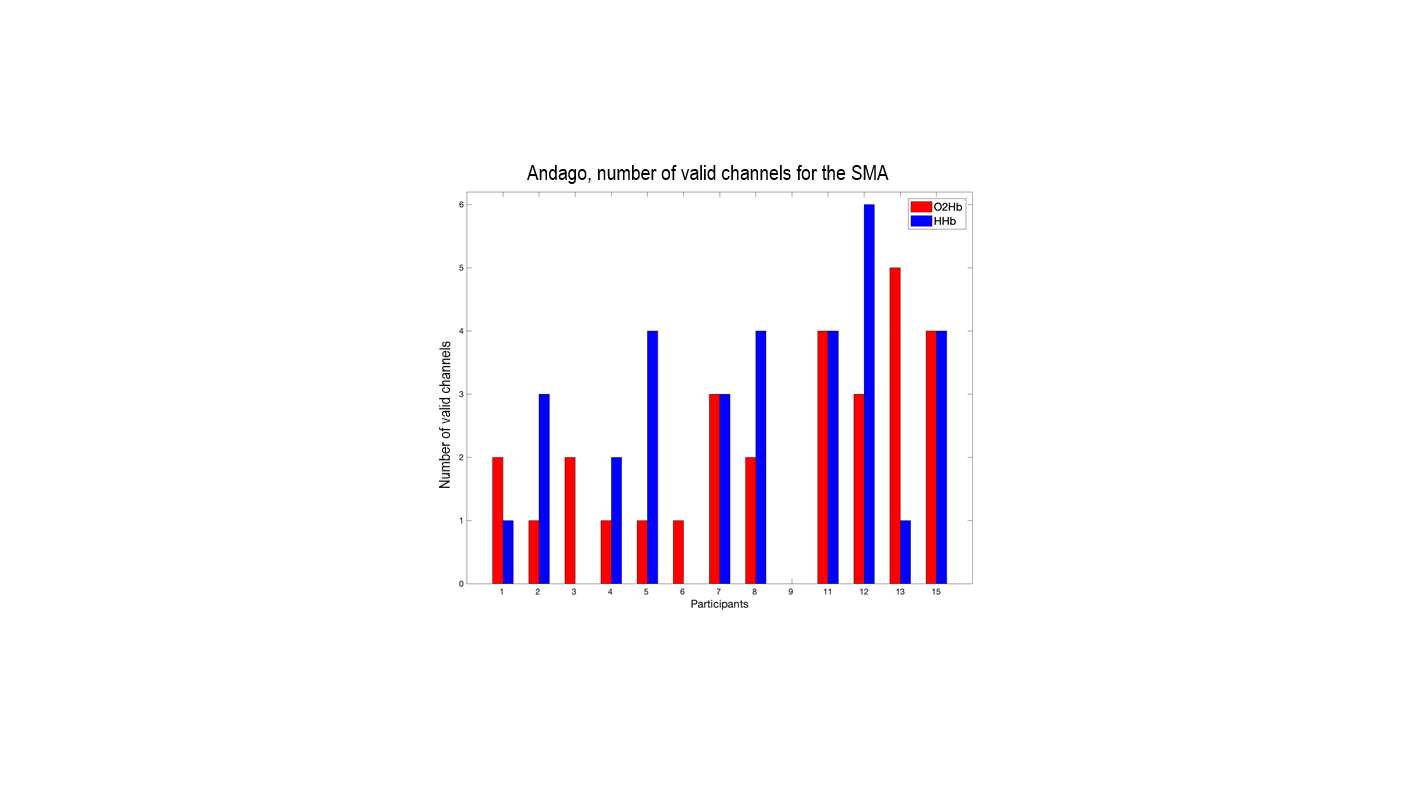


**Supplementary Figure 2.** **Data analysis.**

The figure displays the number of valid channels of each participant for the supplementary motor area (SMA) obtained during Andago walking that can be included in the analysis. In red, oxygenated hemoglobin (O_2_Hb), in blue, deoxygenated hemoglobin (HHb).


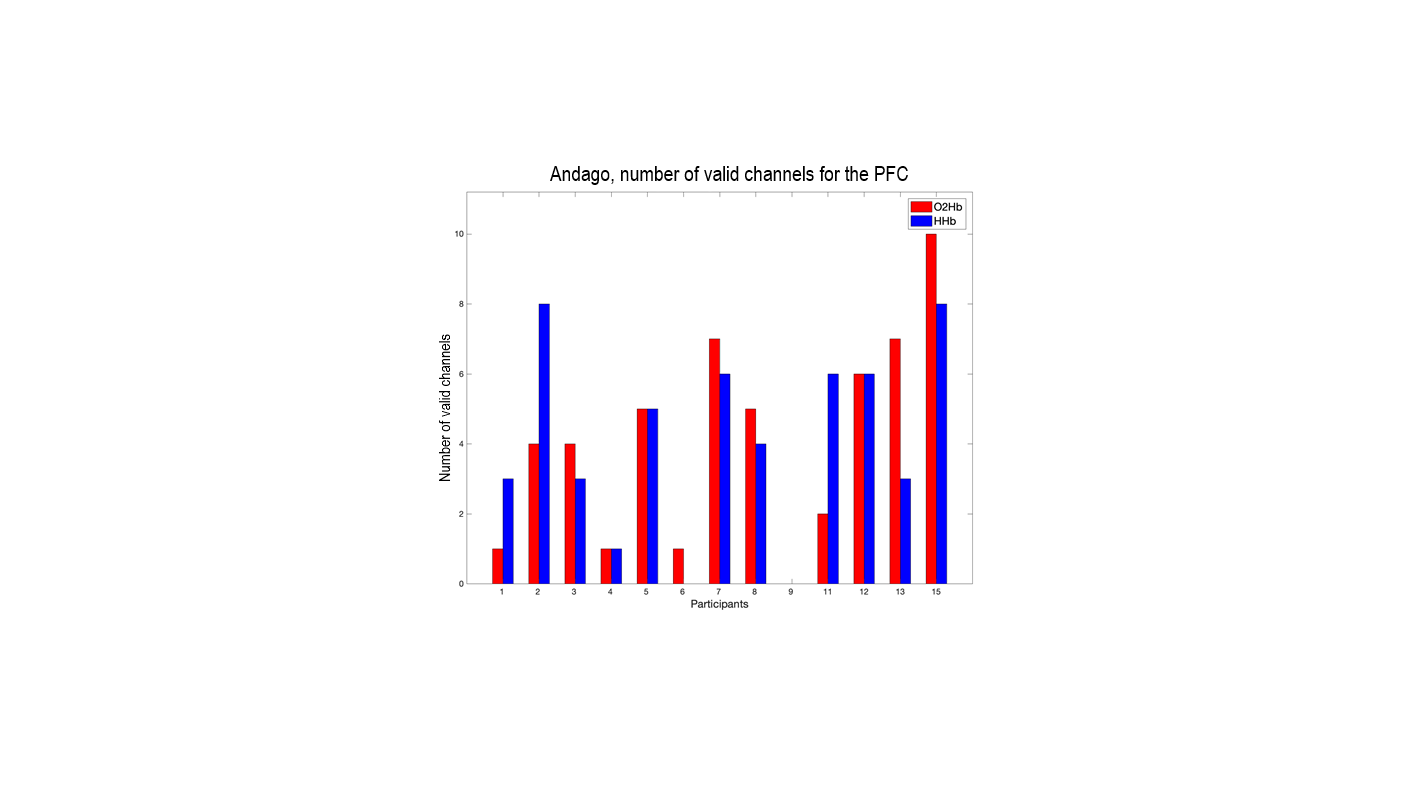


**Supplementary Figure 3. Data analysis.**

The figure displays the number of valid channels of each participant for the prefrontal cortex (PFC) obtained during Andago walking that can be included in the analysis. In red, oxygenated hemoglobin (O_2_Hb), in blue, deoxygenated hemoglobin (HHb).


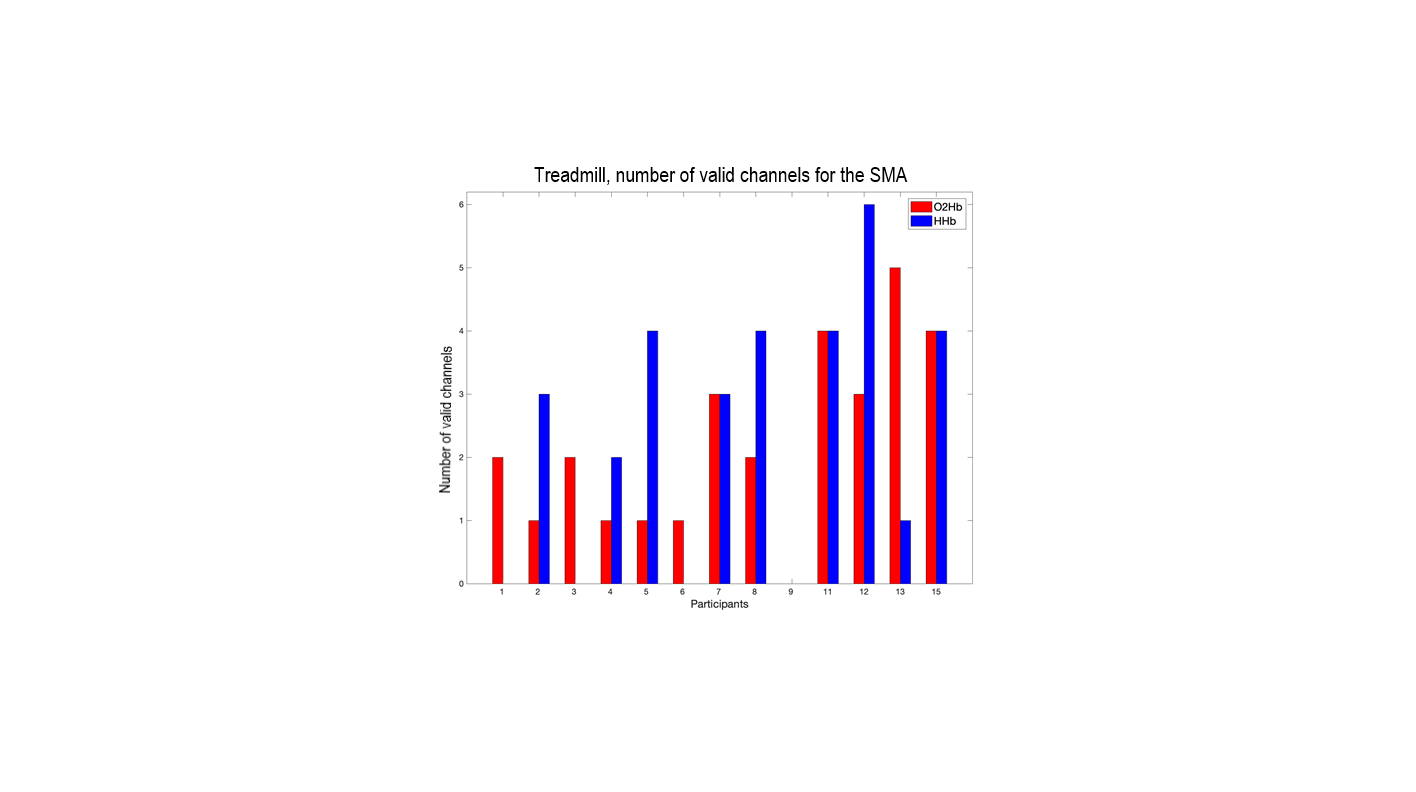


**Supplementary Figure 4.** **Data analysis.**

The figure displays the number of valid channels of each participant for the supplementary motor area (SMA) obtained during treadmill walking that can be included in the analysis. In red, oxygenated hemoglobin (O_2_Hb), in blue, deoxygenated hemoglobin (HHb).


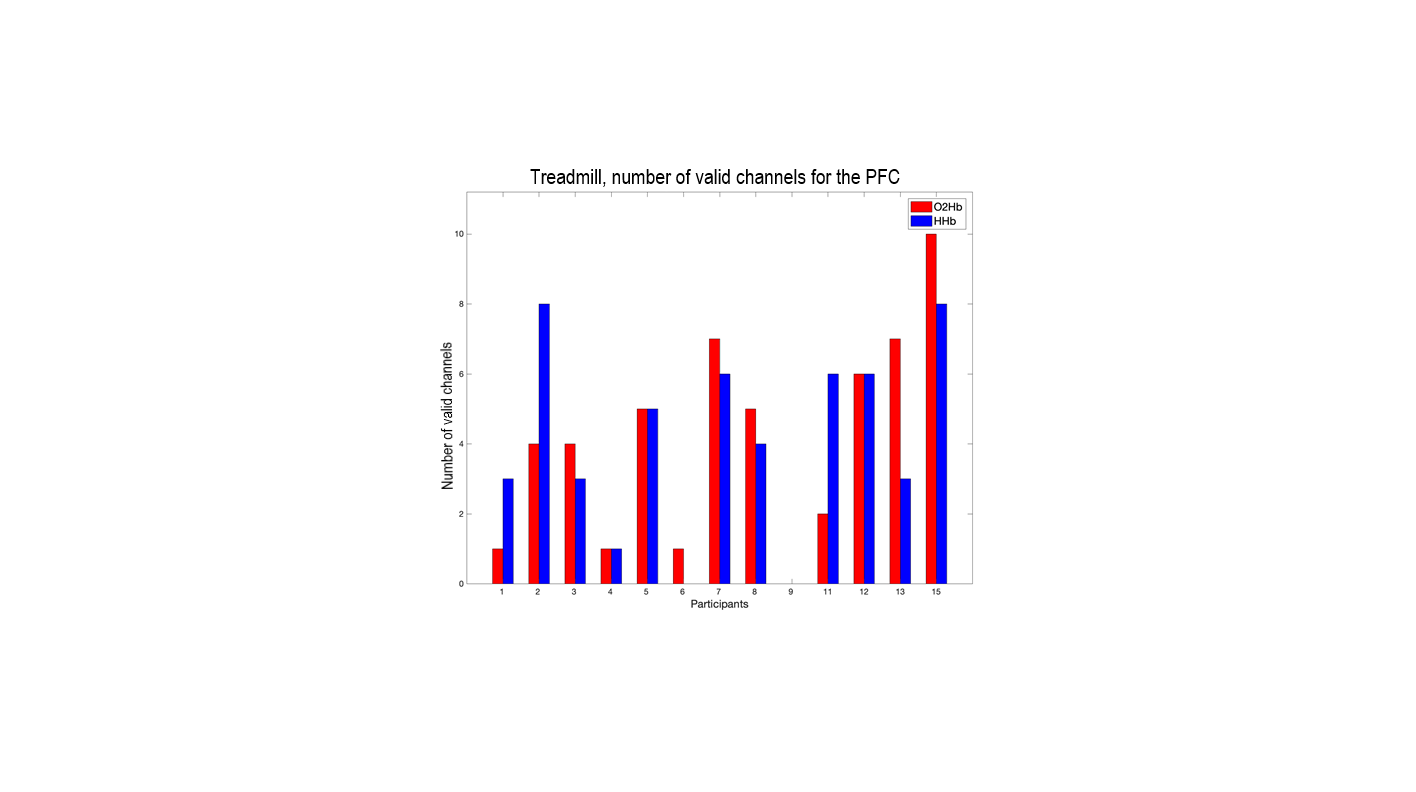


**Supplementary Figure 5.** **Data analysis.**

The figure displays the number of valid channels of each participant for the prefrontal cortex (PFC) obtained during treadmill walking that can be included in the analysis. In red, oxygenated hemoglobin (O_2_Hb), in blue, deoxygenated hemoglobin (HHb).

**
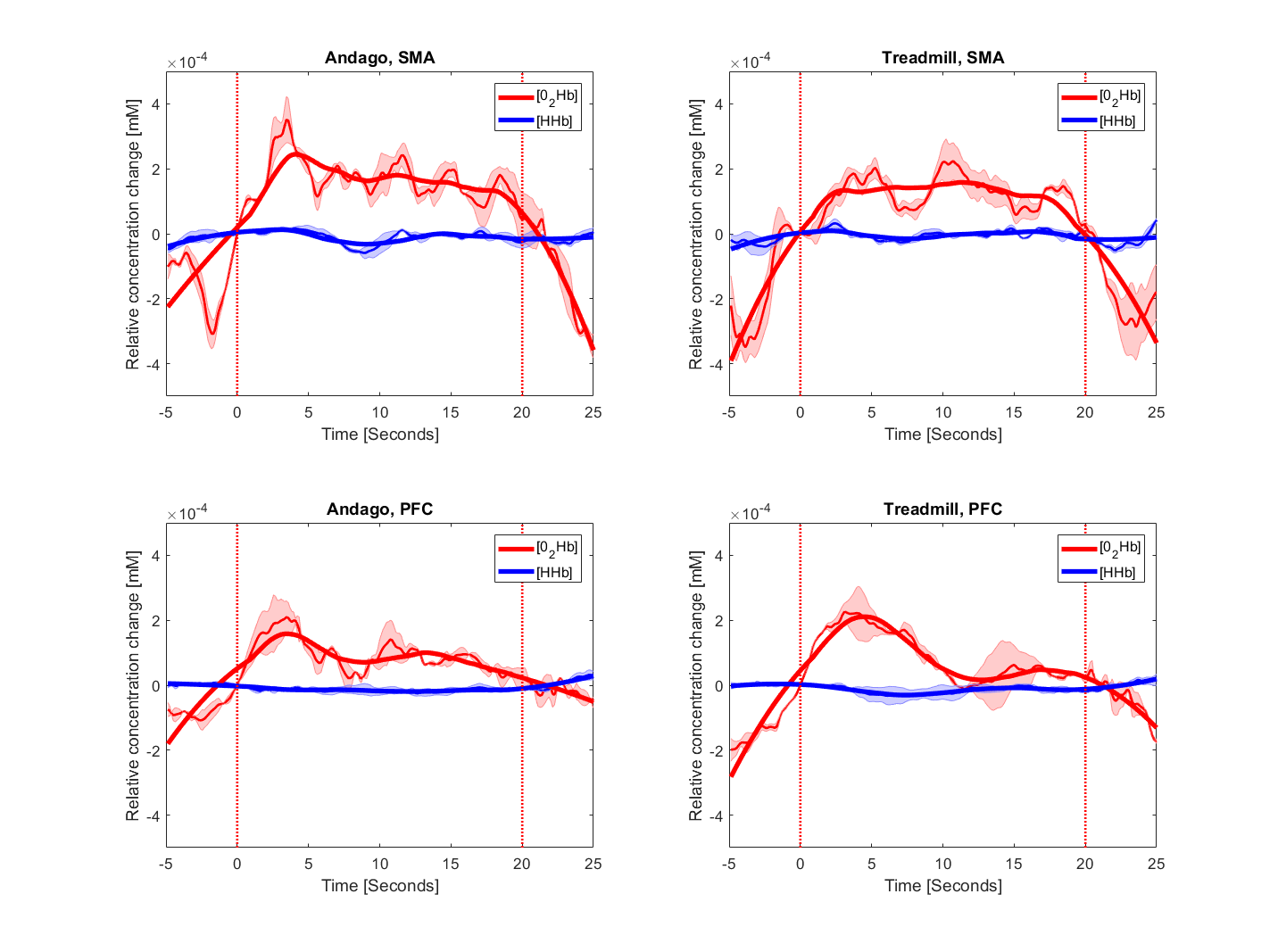
Supplementary Figure 6.** **Averaged time-series for group 1.**

The y-axis shows the concentration changes in millimoles per liter (mM) of O_2_Hb in red and HHb in blue, averaged across the participants with at least one valid channel for the area. Thinner lines represent the averaged curves. The shaded area represents the standard error of the mean. The thick lines represent smoothened concentration changes. The x-axis represents the time of the stimulus (20 s) plus the 5s before and 5s after it. The vertical red lines indicate the onset and end of the stimulus. The plots displaying the hemodynamic responses for the SMA were averaged over 4 participants (SMA: ID 3, 7, 13, and 15) and for the PFC over 3 participants (ID 7, 13, and 15).


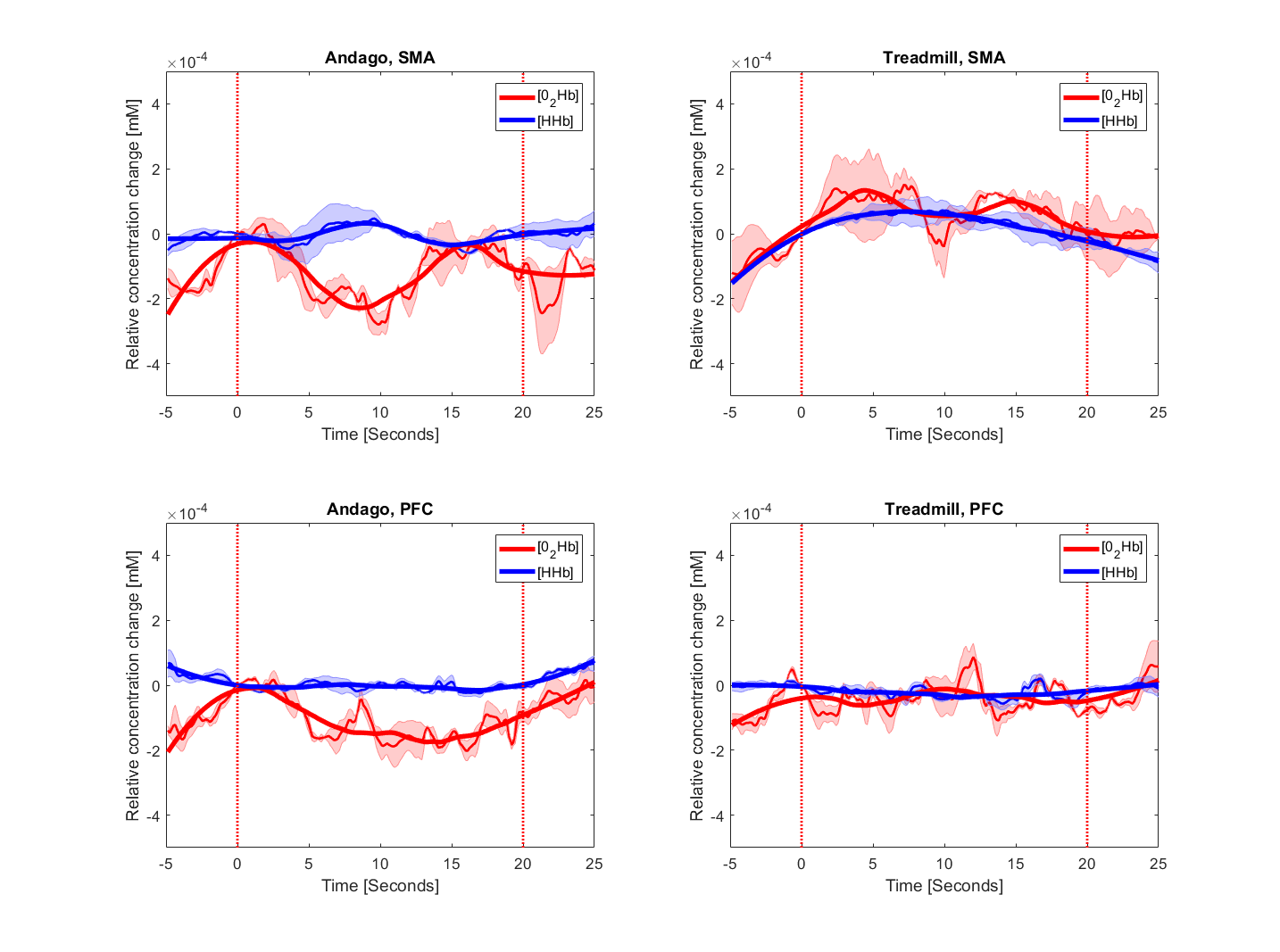


**Supplementary Figure 7. Averaged time-series for group 2.**

The y-axis shows the concentration changes in millimoles per liter (mM) of O_2_Hb in red and HHb in blue, averaged across the participants with at least one valid channel for the area. Thinner lines represent the averaged curves. The shaded area represents the standard error of the mean. The thick lines represent smoothened concentration changes. The x-axis represents the time of the stimulus (20 s) plus the 5s before and 5s after it. The vertical red lines indicate the onset and end of the stimulus. The plots displaying the hemodynamic responses in the SMA were averaged over 2 participants (ID 4 and 5) and averaged over 4 participants for the PFC (ID 1, 3, 4, and 11).


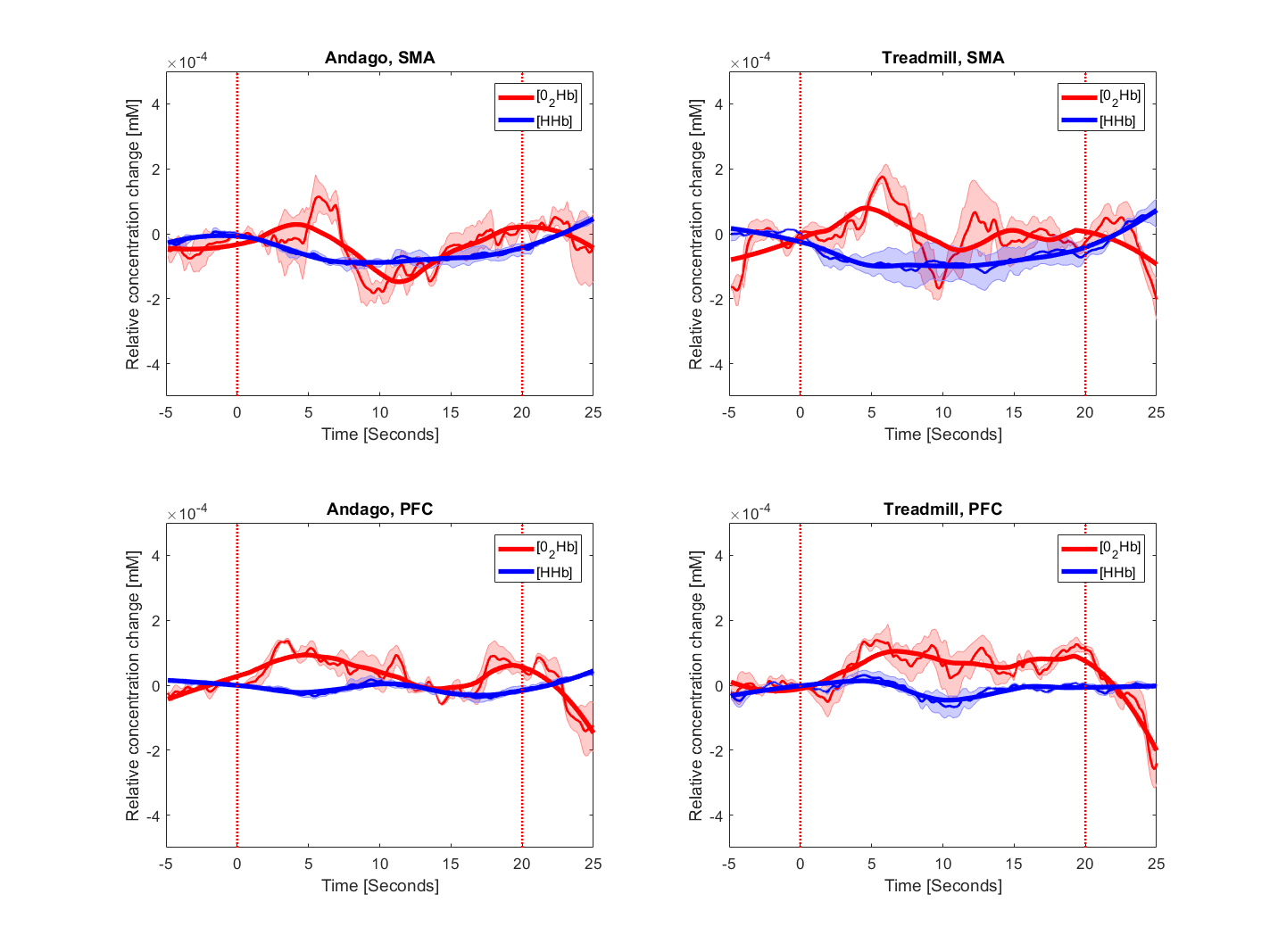


**Supplementary Figure 8.** **Averaged time series for group 3.**

The y-axis shows the concentration changes in millimoles per liter (mM) of O_2_Hb in red and HHb in blue, averaged across the participants with at least one valid channel for the area. Thinner lines represent the averaged curves. The shaded area represents the standard error of the mean. The thick lines represent smoothened concentration changes. The x-axis represents the time of the stimulus (20 s) plus the 5s before and 5s after it. The vertical red lines indicate the onset and end of the stimulus. The plots displaying the hemodynamic responses in the SMA were averaged over 5 participants (SMA: ID 1, 2, 8, 11, and 12) and for the PFC over 4 participants (ID 2, 5, 8, and 12).
